# A Provider-Focused Intervention to Increase Universal HIV Testing among Adolescents in School-Based Health Centers

**DOI:** 10.1007/s10461-024-04444-6

**Published:** 2024-07-24

**Authors:** Neal D. Hoffman, Adam Ciarleglio, Susanna Lesperance-Banks, Tom Corbeil, Harpreet Kaur, Ellen J Silver, Laurie Bauman, Theo G. M. Sandfort

**Affiliations:** 1 Division of Adolescent Medicine, Children’s Hospital at Montefiore, 3415 Bainbridge Avenue, Bronx, NY 10467 USA; 2 Milken Institute School of Public Health, The George Washington University, Washington, DC USA; 3 Montefiore School Health Program, Bronx, NY USA; 4 New York State Psychiatric Institute, HIV Center for Clinical and Behavioral Studies, Columbia University, New York, NY USA; 5 Preventive Intervention Research Center for Child Health, Department of Pediatrics, Albert Einstein College of Medicine, Bronx, NY USA

**Keywords:** Adolescents, HIV testing, School-based health centers

## Abstract

We describe a provider-focused intervention to increase universal HIV testing among adolescent users in a network of School-Based Health Centers (SBHC) and compare the rate of HIV test offer and acceptance for SBHCs with and without the HIV testing intervention. The intervention was implemented at the six largest SBHCs in the 12-site network and included system- and staff-level initiatives, including an implementation coach to support SBHC associates. Rates of HIV test offer and acceptance at six sites in the Intervention Cohort were compared to that at the six sites in a Non-Intervention Cohort which was not randomly selected but had comparable distributions by age, gender and race/ethnicity. The model showed an intervention effect for universal HIV test offer, but no overall effect for test acceptance. Analyzing the intervention effect by whether a patient had a history of sexual activity, the intervention was very effective early in its implementation at increasing test offer to those with no history of sexual activity, and late in its implementation at increasing test acceptance for those with no or unknown sexual activity. Increasing and sustaining universal HIV testing in SBHCs may benefit from using Implementation Science frameworks to guide adaptation of the intervention.

**Supplementary Information:**

The online version contains supplementary material available at 10.1007/s10461-024-04444-6.

## Resumen

Describemos una intervención centrada en proveedores de salud para aumentar las pruebas universales del VIH entre los usuarios adolescentes en una red de Centros de Salud Escolares (SBHC), y comparamos la tasa de oferta y aceptación de pruebas del VIH para los SBHC con y sin la intervención de pruebas del VIH. La intervención se implementó en los seis SBHC más grandes de la red de 12 sitios e incluyó iniciativas a nivel de sistema y de personal, incluido un asesor de implementación para apoyar a los asociados de SBHC. Se compararon las tasas de oferta y aceptación de pruebas de VIH en seis sitios de la cohorte de intervención con las de los seis sitios de una cohorte de no intervención que no fue seleccionada al azar pero que tenía distribuciones comparables por edad, género y raza/etnia. El modelo mostró un efecto de intervención para la oferta universal de pruebas del VIH, pero ningún efecto general para la aceptación de las pruebas. Al analizar el efecto de la intervención en función de si un paciente tenía antecedentes de actividad sexual, la intervención fue muy efectiva al principio de su implementación para aumentar la oferta de pruebas para aquellos sin antecedentes de actividad sexual, y al final de su implementación para aumentar la aceptación de las pruebas para aquellos sin antecedentes o con actividad sexual desconocida. Aumentar y mantener las pruebas universales del VIH en los SBHC puede beneficiarse del uso de marcos científicos de implementación para guiar la adaptación de la intervención.

Youth 13–24 years old accounted for 20% of new HIV infections among adolescents and adults in the U.S in 2019; for new infections among African Americans, youth represented 22% [1]. According to the Centers for Disease Control and Prevention (CDC), comparing differences in estimate of HIV incidence and actual HIV diagnoses, youth 13–24 years old had the highest percentage of undiagnosed HIV infections: 44% compared to 28% of 25–34-year-olds and 15% of 35–44-year-olds [1]. Furthermore, the CDC’s 2019 Youth Risk Behavior Surveillance showed that 90.7% of high school students reported never having taken an HIV test [2]. HIV testing remains critical for establishing an HIV diagnosis, facilitating access to care, and preventing onward transmission.

Since 2003, the CDC has recommended that health care providers offer HIV testing to adolescents and adults regardless of risk, also known as universal testing. In 2013, and reaffirmed in 2019, the U.S. Preventive Services Task Force (USPSTF) recommended screening for HIV infection in adolescents and adults starting at age 15 years, as well as young adolescents at increased risk, with a Grade A level of evidence [3]. This is based on evidence that providers’ risk assessment is often inaccurate; providers underestimate risk for some and overestimate for others [4, 5]. Studies also suggest that patients may feel uncomfortable disclosing risk-behaviors early in the patient-provider relationship [6].

Whereas HIV testing interventions have been successfully implemented in youth in low- and middle-income countries [7, 8], or, within the United Status in targeted high-risk populations such as men who have sex with men and transgender women [9, 10], few interventions have been successful in increasing HIV testing in youth. Several patient-targeted interventions increased testing by providing feedback on self-reported risk behaviors [11–13]. An intervention in a Pediatric Emergency Department demonstrated feasibility in supporting clinical decision-making for offering at-risk-adolescents point-of-care HIV testing [13]. One qualitative study of community health center leaders pointed to provider discomfort and lack of awareness of testing recommendations as barriers to routine testing [14]. An educational intervention to improve provider-initiated HIV testing was modestly successful, maintaining but not increasing testing rates [15]. A pilot of a student-led health marketing campaign in a Chicago Public High School was successful in increasing HIV and Sexually Transmitted Infection testing [16]. School-based health centers (SBHCs) are a strategic site for improving access to HIV testing and prevention for adolescents. SBHCs provide access to primary care, mental, dental, and other health services, serving more than 6.3 million school-age youth in approximately 10% of US public schools, especially youth who are uninsured or under-insured [17–19]. In a recent survey of urban youth of color recruited at community-based organizations, two-thirds indicated they would use school-based HIV testing services if offered confidentially and non-judgmentally [20]. A survey of SBHC providers in New York State, after the NYS Department of Health mandated the offer of HIV testing to all patients ages thirteen and older, showed that, although 71% of SBHCs had on-site testing available, only 41% reported offering testing to eligible patients [21].

We initiated a provider-focused intervention to increase universal HIV testing in a network of urban SBHCs serving adolescents. The purpose of this analysis is to compare the rate of HIV test offers and HIV test acceptance for adolescent patients in SBHCs with and without the HIV testing intervention.

## Methods

### Participants

The study took place at six SBHCs in public high school campuses in the Bronx, New York. The Bronx, with a population of 1.47 million residents and a median household income of $35,302, ranks 62nd of 62 counties for all health indicators. 43.7% of the population is Black, 56.2% is Latino; 24.9% is under 18 years old.

The SBHCs, affiliated with a large non-profit academic medical center, are staffed by on-site teams providing co-located medical, behavioral, dental, health outreach and education services. Each team consists of one to two medical providers (physician or nurse practitioner), a nurse or patient-care technician (PCT), a mental health provider, a dentist, front desk clerk, outreach representative and health educator. Parent/guardian consent is required for students under age 18 years of age. However, students may self-enroll for confidential reproductive/sexual health services only, including STI testing and treatment and Contraception. All reproductive/sexual health services are confidential according to state health regulations. Services at the SBHC require no out-of-pocket expenses for families.

For this intervention, we selected the six largest SBHC sites in the network, each with three to six high schools, and created two sets of three SBHC sites to start the same intervention one year apart: one initiated the intervention in Fall 2016, the second in the Fall 2017. The six sites were designated as the Intervention (I) Cohort.

### Procedure

The Universal HIV Testing intervention aimed to integrate a routine offer of HIV testing at annual preventive visits and at initial and follow-up reproductive and sexual health visits. The standardized procedure including a first offer being made by the nurse (or PCT) completing the visit intake with vital signs at the start of the visit and before asking about sexual and substance use activity. If the patient accepted the offer to test, the nurse (or PCT), under a standardized existing order set, obtained the oral specimen and initiated specimen processing within the on-site lab. If the patient declined the offer, the nurse informed the provider who, upon initiating the visit, was trained to re-offer the test in a non-coercive, non-judgmental manner. OraSure Technologies’ Third Generation Rapid Oral Test was utilized as a general procedure at the SBHCs due to limited use of routine lab testing at the SBHCs and the strong appeal to students for oral testing.

The intervention included system- and staff-level initiatives. System-level initiatives included: (1) practice workflows for offering HIV testing, developed with input from SBHC staff; workflow innovations included adjustments to communication among health center staff, especially the front desk staff, nurse or PCT and the medical providers; (2) use of performance improvement tools (Process Maps and Plan-Do-Study-Act cycles) [22]; and (3) enhancement of patient messaging about testing in poster format and on electronic monitors in the waiting areas, emphasizing the message that HIV testing is part of routine health care and is offered to all users of the SBHC.

Staff-level initiatives included (1) twice annual trainings; (2) a one-time small-group skills building workshop in the first intervention year around verbal scripts to offer HIV testing and prevention counseling, as well as to communicate positive HIV test results, provide immediate support, often with the participation of the on-site mental health provider, and to facilitate linkage to HIV care through the HIV treatment program within the larger Medical Center; (3) optimization of EHR tools to benefit associates in the accurate and efficient viewing and documenting; (4) “elbow support” to provide technical assistance to associates for accurate and efficient documentation in the Electronic Health Record (EHR); (5) visual aids at workstations to further assist and remind associates about accurate documentation in the EHR; and (6) team incentives for exceeding monthly targets, such as catered lunches and coffee supplies.

For all six teams, a full-time Implementation Coach attended monthly team meetings and made episodic site visits for one-on-one interactions to provide data feedback, and technical and emotional support, and get input from staff, to improve associate competence, confidence and motivation.

### Instrumentation

Rates of HIV testing at the I Cohort were compared to rates of six other Bronx SBHCs within the same network that did not receive the intervention, here designated as the Non-Intervention (NI) Cohort. The sites in the Non-Intervention Cohort were not randomly selected but had comparable distributions by age, gender and race/ethnicity. The six NI sites also used the OraSure Technologies’ Third Generation Rapid Oral Test. These SBHC teams were not guided or supported in any workflow efforts focused on universal HIV-testing, did not receive monthly data feedback, nor any support from the Implementation Coach; however, all SBHCs had available a system-wide “Best Practice Advisory” alert in the EHR regarding annual HIV testing.

We collected data through extraction from the Electronic Health Record on all medical visits in the pre-intervention (baseline) year through the 2018/2019 school year for the I and NI Cohorts. Extracted data included age, gender, race/ethnicity, ever sexually active, whether an HIV test was offered during the school year, and if so, whether the offer was accepted. We also included data from the 2019/2020 school year which we designated as a post-intervention year during which no Implementation Coach was available. This final year was truncated because of schools being closed in March 2020 due to COVID-19. For each school year, we defined last medical visit within the school year as the Index Event and looked for the most recent HIV Test or Test Offer on or within 12 months of the Index Event to satisfy the outcomes. We defined “Sexually Active Ever” (SAE) by looking for the most recent response (Yes, No, Unknown) on or prior to the Index Event.

Medical providers documented at each visit using a structured field in the EHR if HIV Testing was offered and if the patient accepted or declined. To detect if providers were overreporting the HIV Test Offer, we periodically did random exit interviews with patients at the I Cohort sites asking if the provider had offered an HIV Test to assess agreement with provider documentation; no overstatement of offers was detected.

## Data Analysis

Clinical and demographic information were summarized for each cohort. Means and standard deviations were provided for age while frequencies and proportions are provided for other demographic variables and for HIV test offer and acceptance rates.

To assess the effects of the intervention, we fit logistic mixed effects models with either HIV test offer or acceptance as the outcomes. Models included a facility-specific random intercept and facility-specific random slope for schoolyear to account for clustering by SBHC. A patient-specific random intercept was also included to account for multiple visits for patients in more than one school year. Each model included the following covariates: age (centered and scaled), gender, race, ethnicity, school year (as a factor variable to account for secular trends), sexually active ever (SAE) status, and years since initiation of Universal HIV Testing intervention. From this model, we computed intervention effect estimates at 1, 2, 3, and 4 years after initiation. We also fit models that included interaction between SAE status and years since initiation of the intervention to investigate whether the effect of the intervention differed by SAE status. To fit the logistic model with HIV test acceptance as the outcome, the sample was restricted to only those who were offered an HIV test. The intervention effects estimated from these models are presented as patient/SBHC-specific odds ratios (ORs). We also report the corresponding 95% confidence interval (CI) for each OR. All analyses were conducted in R version 4.0.2 [23]. Mixed effects models were fit using functions from the lme4 package and likelihood ratio tests were conducted using functions from the lmerTest package [24, 25]. All test statistics reported below are Wald ${\chi }^{2}$ test statistics with the null distribution being ${\chi }^{2}$ with 1 degree of freedom (df) unless specified otherwise.

## Results

### Sample

Across all study years, there were 27,328 patients in the Intervention (I) Cohort and 18,025 patients in the Non-Intervention (NI) Cohort. Demographic and clinical information are shown in Table 1 for the baseline year 2015–2016 only since the demographics did not differ meaningfully across years for either cohort.

**Table 1:** Clinical and demographic information for visits at baseline. (baseline for the three early sites in the intervention cohort is the 2015–2016 school year and for the three delayed sites in the intervention cohort is the 2016–2017 school year. Baseline for the non-intervention cohort is taken as the 2015–2016 school year.)

	Intervention	Non-Intervention
n	5712	3582
Age (mean (SD))	15.95 (1.47)	16.34 (1.59)
Gender (Male) (%)	2410 (42.2)	1420 (39.6)
Ethnicity (%)		
Non-Span/Hisp/Lat	1897 (33.2)	1181 (33.0)
Span/Hisp/Lat	2835 (49.6)	1592 (44.4)
Unknown	980 (17.2)	809 (22.6)
Race (%)		
White	215 (3.8)	134 (3.7)
Black/Afr. Am.	1573 (27.5)	1066 (29.8)
Other	3211 (56.2)	1729 (48.3)
Unknown	713 (12.5)	653 (18.2)
Sexually Active Ever (%)		
No	2753 (48.2)	1343 (37.5)
Yes	2146 (37.6)	1129 (31.5)
NA	813 (14.2)	1110 (31.0)

### HIV Test Offer

**Fig. 1:**
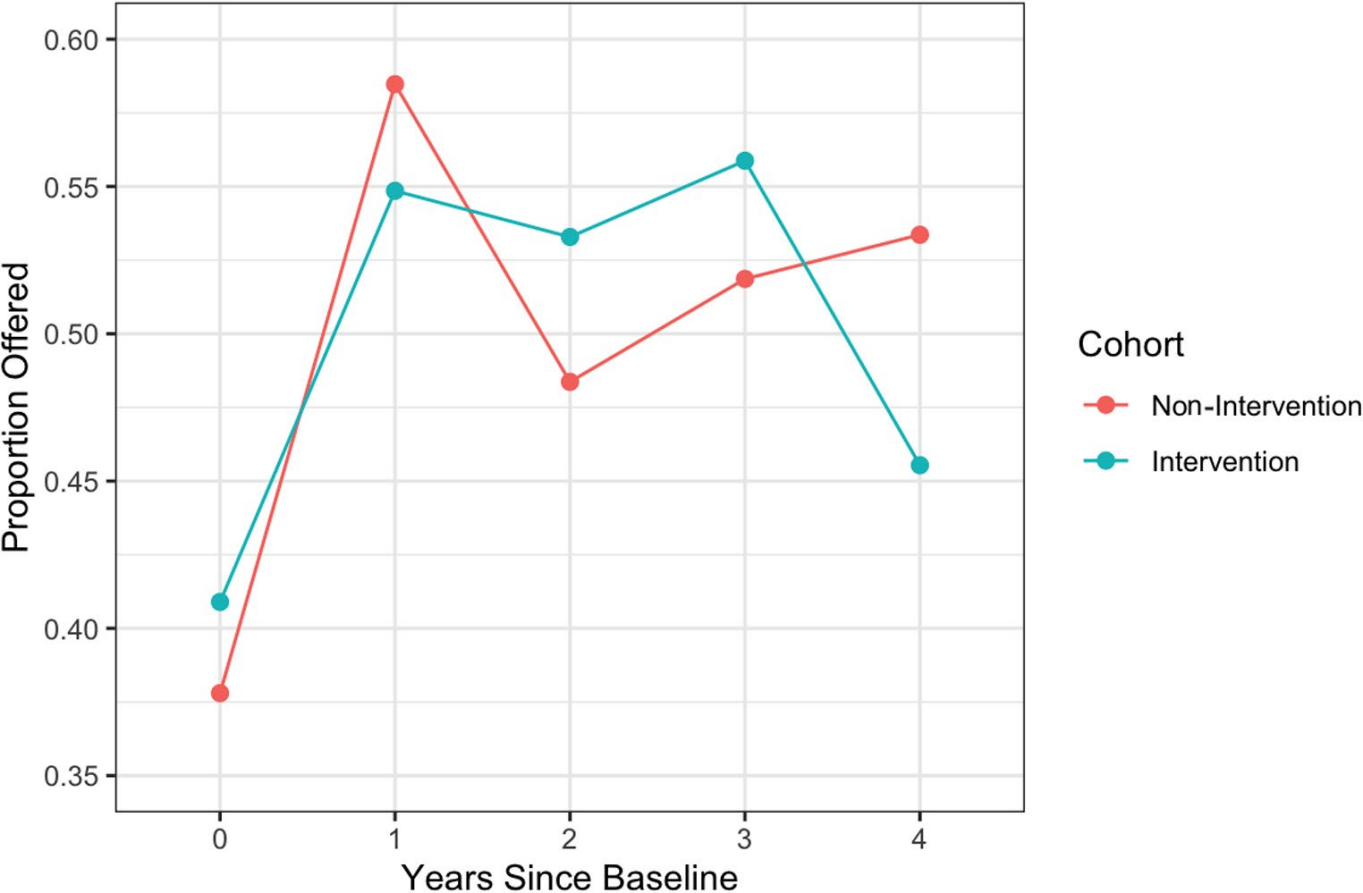
Proportion of patients who received an HIV test offer among those with at least one medical visit in a school year stratified by cohort. The Intervention Cohort includes data from both the early and delayed intervention groups combined (where Year 0 corresponds to 2015–2016 for the early group and to 2016–2017 for the delayed group). Year 0 for the Non-Intervention Cohort is taken as 2015–2016

**Fig. 2:**
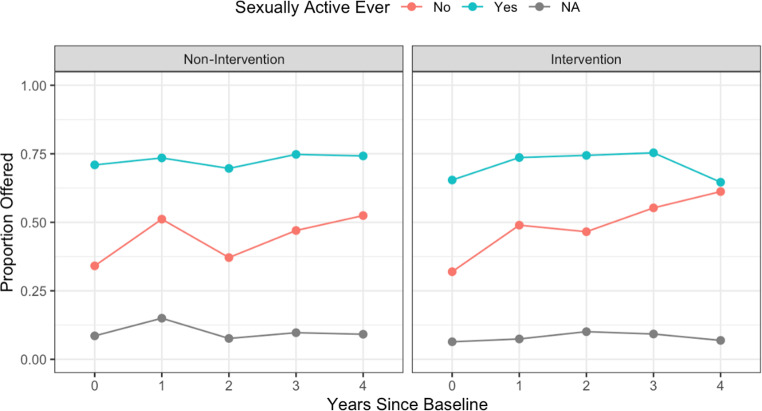
Proportion of patients who received an HIV test offer among those with at least one medical visit in a school year stratified by cohort and sexually active ever status. The Intervention Cohort includes data from both the early and delayed intervention groups combined (where Year 0 corresponds to 2015–2016 for the early group and to 2016–2017 for the delayed group). Year 0 for the Non-Intervention Cohort is taken as 2015–2016

The HIV test offer rates for the I and NI Cohorts (Fig. 1) in their baseline years were 41%, and 38%, respectively. The offer rate for the I Cohort increased from 41 to 55% in the first intervention year, whereas for the NI Cohort the rate increased from 38 to 59%. The I Cohort sustained those gains over the next two years (53% and 56%), but then dropped to 46% in the fourth intervention year, although still above the baseline rate. The NI Cohort dropped somewhat in Years 2 and 3 and 4 (48% and 51% and 53%), less than the Year 1 gain but still above baseline. HIV test offer rates were highest in both cohorts for patients who were ever sexually active, and lowest for those for whom no SAE status was available. (Fig. 2)

The overall intervention effect was strongest in Years 1 and 2, but then dropped in Years 3 and 4. There is strong evidence that SAE status modifies the effect of the intervention on the HIV test offer rate (likelihood ratio test statistic ${\chi }^{2}$ = 274.75, df = 8, *p* < 0.001). Specifically, we found that among those who were never sexually active, the effect of the intervention was statistically significant in years 1 (OR = 1.81, 95% CI 1.38–2.38, ${\chi }^{2}$ = 18.31, *p* < 0.001), 2 (OR = 1.72, 95% CI 1.34–2.22, ${\chi }^{2}$ = 17.67, *p* < 0.001), and 3 (OR = 1.71, 95% CI 1.28–2.29, ${\chi }^{2}$ = 13.11, *p* < 0.001) after initiation. In year 4, the effect size remained the same but did not reach significance (OR = 1.82, 95% CI 0.97–3.42, ${\chi }^{2}$ = 3.48, *p* = 0.062). There was no evidence of intervention effects in any years after initiation of the intervention either among those who were ever sexually active or among those whose sexual activity status was unknown, with one exception. Among those who were ever sexually active, we observed a decrease in the odds of receiving an HIV test offer in the 4th year after initiation relative to baseline (OR = 0.38, 95% CI 0.21–0.72, ${\chi }^{2}$ = 9.01, *p* = 0.003) (Table 2).

**Table 2:** Universal HIV Testing intervention effect on HIV test offer after 1–4 years overall and stratified by sexually active ever (SAE) status

	Year 1	Year 2	Year 3	Year 4
Overall	1.39 (1.06, 1.81) ${\chi }^{2}$ = 5.82, *p* = 0.016	1.39 (1.08, 1.77) ${\chi }^{2}$ = 6.80, *p* = 0.009	1.15 (0.88, 1.51) ${\chi }^{2}$ = 1.06, *p* = 0.304	0.73 (0.41, 1.32) ${\chi }^{2}$ = 1.07, *p* = 0.302
SAE = NO	1.81 (1.38, 2.38) ${\chi }^{2}$ = 18.31, *p* < 0.001	1.72 (1.34, 2.22) ${\chi }^{2}$ = 17.67, *p* < 0.001	1.71 (1.28, 2.29) ${\chi }^{2}$ = 13.11, *p* < 0.001	1.82 (0.97, 3.42) ${\chi }^{2}$ = 3.48, *p* = 0.062
SAE = YES	1.09 (0.83, 1.44) ${\chi }^{2}$ = 0.39, *p* = 0.531	1.12 (0.86, 1.45) ${\chi }^{2}$ = 0.73, *p* = 0.392	0.80 (0.60, 1.07) ${\chi }^{2}$ = 2.28, *p* = 0.131	0.38 (0.21, 0.72) ${\chi }^{2}$ = 9.01, *p* = 0.003
SAE = UNK	0.98 (0.65, 1.47) ${\chi }^{2}$ = 0.01, *p* = 0.910	1.36 (0.96, 1.94) ${\chi }^{2}$ = 2.99, *p* = 0.084	0.91 (0.63, 1.31) ${\chi }^{2}$ = 0.27, *p* = 0.601	0.51 (0.26, 1.02) ${\chi }^{2}$ = 3.26, *p* = 0.057

First row for each category corresponds to OR (95% CI) for receiving an HIV test offer comparing 1–4 years of intervention vs. 0 years

Age (OR = 1.03 for each 1.5-year increase, 95% CI 1.01–1.06, ${\chi }^{2}$ = 6.04, *p* = 0.014), male sex (OR = 0.71 compared to female sex, 95% CI 0.68–0.74, ${\chi }^{2}$ = 208.03, *p* < 0.001), black/African American race (OR = 1.16 compared to white, 95% CI 1.01–1.33, ${\chi }^{2}$ = 4.23, *p* = 0.040), unknown race (OR = 1.17 compared to white, 95% CI 1.02–1.34, ${\chi }^{2}$ = 4.71, *p* = 0.030), unknown ethnicity (OR = 1.09 compared to non-Hispanic, 95% CI 1.00–1.18, ${\chi }^{2}$ = 4.15, *p* = 0.042), were independently associated with HIV test offer, based on the fully adjusted model. (Data not provided in the Tables.)

### HIV Test Acceptance after Offer

**Fig. 3:**
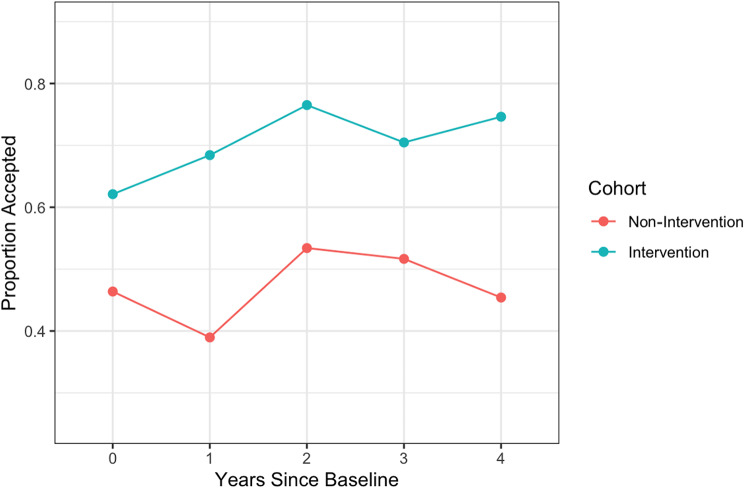
Proportion of patients accepting an HIV test offer among those with at least one offer in a school year by cohort. The Intervention Cohort includes data from both the early and delayed intervention groups combined (where Year 0 corresponds to 2015–2016 for the early group and to 2016–2017 for the delayed group). Year 0 for the Non-Intervention Cohort is taken as 2015–2016

The HIV test acceptance rates for the I and NI Cohorts in their baseline years were 62% and 46%, respectively. The test acceptance rate for the I Cohort increased from 62 to 68% in the first intervention year, whereas the NI Cohort decreased from 46 to 39%. By the end of Year 4, the I Cohort showed a 75% acceptance rate, whereas the NI Cohort showed a 45% acceptance rate. (Fig. 3)

**Fig. 4:**
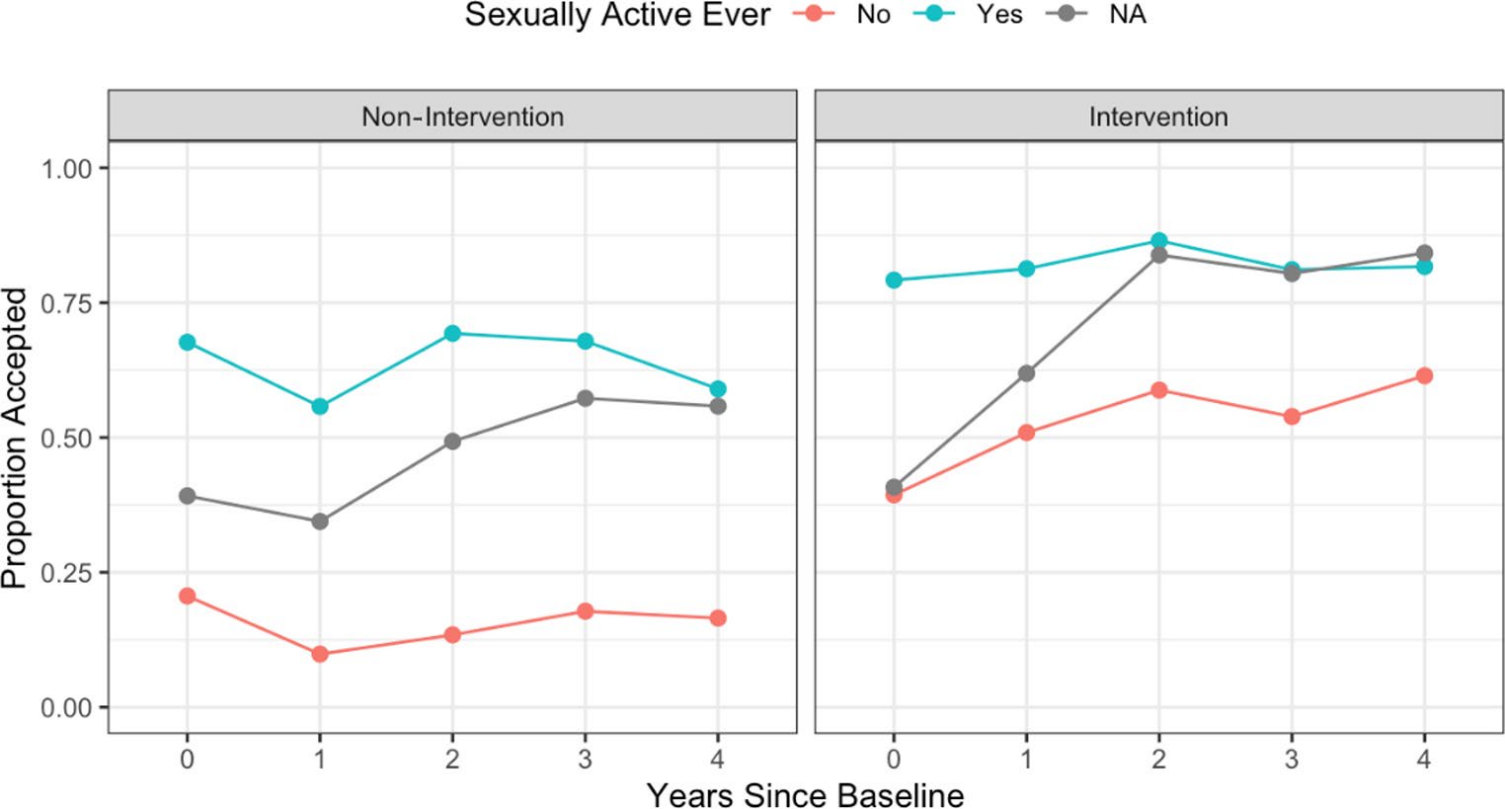
Proportion of patients accepting an HIV test offer among those with at least one offer in a school year stratified by cohort and sexually active ever status. The Intervention Cohort includes data from both the early and delayed intervention groups combined (where Year 0 corresponds to 2015–2016 for the early group and to 2016–2017 for the delayed group). Year 0 for the Non-Intervention Cohort is taken as 2015–2016

For both Cohorts, HIV test acceptance was highest for patients who were ever sexually active, and lowest for those who had never been sexually active. HIV test acceptance rates remained stable in both cohorts for those who were ever sexually active, whereas for those never sexually active, rates increased from 39 to 62% in the I Cohort, with no increases seen for the NI Cohort. For patients with unknown SAE status, test acceptance rates increased for both cohorts from the baseline year although the I Cohort sustained greater increases. (Fig. 4)

There was no evidence of an overall intervention effect in any years after initiation of the intervention. There was strong evidence that SAE status modified the effect of the intervention on the HIV test acceptance rate (likelihood ratio test statistic ${\chi }^{2}$ = 103.43, df = 8, *p* < 0.001). Among those who were never sexually active, the effect of the intervention was statistically significant in years 3 (OR = 5.57, 95% CI 1.34–23.19, ${\chi }^{2}$ = 5.58, *p* = 0.018) and 4 (OR = 7.88, 95% CI 1.89–32.82, ${\chi }^{2}$ = 8.03, *p* = 0.005) after initiation of the intervention. A similar pattern held among those with unknown SAE status with evidence of a statistically significant intervention effect in years 3 (OR = 7.29, 95% CI 1.59–33.29, ${\chi }^{2}$ = 6.56, *p* = 0.010) and 4 (OR = 11.85, 95% CI 2.42–58.06, ${\chi }^{2}$ = 9.30, *p* = 0.002) after initiation of the intervention. There was no evidence of an intervention effect among those who had ever been sexually active (Table 3).

**Table 3:** Universal HIV Testing intervention effect on HIV test acceptance after 1–4 years overall and stratified by sexually active ever (SAE) status

	Year 1	Year 2	Year 3	Year 4
Overall	1.25 (0.62, 2.52) ${\chi }^{2}$ = 0.40, *p* = 0.528	1.79 (0.48, 6.64) ${\chi }^{2}$ = 0.75, *p* = 0.386	3.26 (0.69, 15.33) ${\chi }^{2}$ = 2.24, *p* = 0.134	4.37 (0.94, 20.39) ${\chi }^{2}$ = 3.53, *p* = 0.060
SAE = NO	1.8 (0.85, 3.81) ${\chi }^{2}$ = 2.33, *p* = 0.127	2.65 (0.72, 9.76) ${\chi }^{2}$ = 2.14, *p* = 0.143	5.57 (1.34, 23.19) ${\chi }^{2}$ = 5.58, *p* = 0.018	7.88 (1.89, 32.82) ${\chi }^{2}$ = 8.03, *p* = 0.005
SAE = YES	1.01 (0.48, 2.13) ${\chi }^{2}$ = 0.00, *p* = 0.973	1.62 (0.44, 5.95) ${\chi }^{2}$ = 0.53, *p* = 0.467	2.53 (0.61, 10.49) ${\chi }^{2}$ = 1.64, *p* = 0.200	2.64 (0.64, 10.96) ${\chi }^{2}$ = 1.78, *p* = 0.182
SAE = UNK	0.99 (0.39, 2.53) ${\chi }^{2}$ = 0.00, *p* = 0.988	4.03 (0.98, 16.59) ${\chi }^{2}$ = 3.72, *p* = 0.054	7.29 (1.59, 33.29) ${\chi }^{2}$ = 6.56, *p* = 0.010	11.85 (2.42, 58.06) ${\chi }^{2}$ = 9.30, *p* = 0.002

First row for each category corresponds to OR (95% CI) for accepting an HIV test offer comparing 1–4 years of intervention vs. 0 years

Male sex (OR = 0.74 compared to female sex, 95% CI 0.69–0.80, ${\chi }^{2}$ = 64.87, *p* < 0.001), black/African American race (OR = 1.55 compared to white, 95% CI 1.25–1.92, ${\chi }^{2}$ = 16.21, *p* < 0.001), other race (OR = 1.26 compared to white, 95% CI 1.03–1.55, ${\chi }^{2}$ = 5.16, *p* = 0.023), Spanish/Hispanic/Latino ethnicity (OR = 1.13 compared to non-Hispanic, 95% CI 1.01–1.28, ${\chi }^{2}$ = 4.40, *p* = 0.036), unknown ethnicity (OR = 1.20 compared to non-Hispanic, 95% CI 1.06–1.36, ${\chi }^{2}$ = 8.08, *p* = 0.004) were significantly associated with HIV test acceptance. (Data not provided in the Tables.)

## Discussion

This is the first multi-component provider-focused intervention to increase HIV testing in urban SBHCs in the United States among a general population of in-school youth. We were able to demonstrate an intervention effect for HIV test offer but not for test acceptance. Notably, offer rates in the Intervention (I) Cohort increased for all three years above the respective baselines. However, the I Cohort was not able to sustain improvement in the final intervention year when support and data feedback via the Implementation Coach stopped.

There was strong evidence that SAE status modified the effect of intervention. Among patients who were never sexually active, HIV test offer and HIV test acceptance rates increased from baseline in the I Cohort, in contrast to those who were sexually active ever and those for whom sexually active status was unknown. This suggests that our efforts to train the SBHC teams around the importance of the universal, or non-risk-based, offer of HIV testing in this setting, as per CDC recommendations, was successful. This is based on the possibility that youth may not disclose their sexual or other risk behaviors to the clinician. The limited effect on testing for those who were sexually active may reflect the already relatively high rates of HIV testing observed for sexually active youth at the start of the intervention.

Both outcomes were associated with various demographic characteristics. There was a significant association of older age for test offer, although not for test acceptance. This increased offer may be explained by providers’ perception that older youth are more likely to be at risk, and more developmentally and socially able to perceive their risk [26]. Male youth were less likely to be offered and to accept a test. This is consistent with YRBS findings that showed high school males were less likely to be HIV tested [2]. Many studies also suggest a low uptake of preventive sexual health services among adolescent males because of lower participation in routine care [27]. And, finally tests were more likely to be offered to and accepted by black/African American youth and youth of unknown race and unknown ethnicity, compared to white patients, consistent with YRBS findings that showed that youth of color were slightly more likely to be HIV tested than white youth [2]. Whereas this outcome may reflect providers efforts to address health inequities for people of color related to HIV prevention and treatment [28], it may also represent implicit bias on the part of providers.

That we did not show an overall intervention effect on acceptance of the HIV test by the youth might be explained by the fact that compared to the offer, acceptance is a more complex behavior that both provider and patient play a role in. Our intervention focused on changing provider behavior through trainings and on-site support to increase skills and motivation, as well as through visual aids at the workstation and within the EHR itself to support provider decision-making. Efforts to affect patient behavior around testing were limited in the intervention. Early in the intervention, all sites in the I Cohort received small-group training to practice counseling with the goal of increasing patient acceptance of the test offer. The absence of an effect may suggest the need to amplify these types of small-group trainings over time to improve provider impact on patient acceptance. It is also possible that youth who disclose being sexually active perceive their HIV risk and are concerned about stigma and confidentiality [29].

Finally, despite some gains in both outcomes, the effects were not sustained once support and data feedback by the Implementation Coach stopped. This may reflect that only the three SBHCs that started the intervention first were eligible to have four years of program implementation, as well as the fact that the last year was truncated by service shutdown forced by the COVID-19 outbreak. Nevertheless, a recent systematic review of interventions to increase HIV testing (in varied settings) identified intrapersonal factors as important barriers, and institutional factors as important facilitators [30], suggesting the need for a greater involvement on the part of institutional leadership. One might question if the magnitude of the observed effect warrants the intensive effort that went into this intervention; without a cost-effectiveness analysis, it is hard to say. However, the public health imperative to increase HIV testing, and therefore access to care, remains.

## Limitations

The study findings may not be generalizable to all care settings, and perhaps not to SBHCs without the same resources or in non-urban settings. The study is limited by the fact that we cannot distinguish among the different components of the intervention to know which impacted the rates of test offer and acceptance most. Another limitation is that providers may have omitted documentation of their having offered the HIV test; if this is the case, we may be underestimating the offer rate. In terms of HIV test acceptance, we lack the data to be able to make distinctions between provider and patient contributions to this outcome. Finally, although this intervention did not include HIV test offers at non-preventive care visits, that is, acute or sick visits, we included in our analysis all patients regardless of visit purpose because our EHR did not assign structured data to distinguish acute versus preventive visits at the SBHC. Given this, we may be underestimating the impact of the intervention on HIV preventive services. We are also unable to confirm if there was any contamination to the NI Cohort sites through staff floating and through shared access to HIV-test decision-making tools in the EHR.

## Conclusions

This study confirms that school-based health centers present a strategic opportunity to improve access to testing. However, in order to reproduce a complex intervention like this in other SHBC networks, one may benefit from more optimal utilization of conceptual implementation science frameworks. Systematically identifying barriers and facilitators would then help with adjustments to workflows and procedures as the intervention proceeds. A preliminary analysis of this intervention using the Conceptual Framework for Implementation Research in the second year of the intervention suggested that available resources such as consistent staffing and leadership support to teams to manage competing priorities may be as important as the primary elements of the intervention itself [31]. In addition, qualitative inquiry about the experience of the associates participating in the intervention as well as about the role of the implementation coach and the tools utilized to assist the teams in building on and sustaining their gains would also be elucidating. Youth input into implementation science research around adolescent HIV testing, and efforts to improve overall youth engagement remains paramount and needs further attention [32]. Evidence for the effect of practice change interventions on the broader range of preventive care by primary care clinicians remains elusive; and more work is needed to understand how to implement more effective provider-based interventions [33, 34]. A recent conceptual analysis points to the importance of using an ongoing iterative process at multiple levels to effectively change practice [35].

## Electronic Supplementary Material

Below is the link to the electronic supplementary material.

Supplementary Material 1
