# Supplementary material for: A Provider-Focused Intervention to Increase Universal HIV Testing among Adolescents in School-Based Health Centers
Source: AIDS Behav. 2024 Jul 24;28(11):3596–604. doi: 10.1007/s10461-024-04444-6 (PMC11471375; doi:10.1007/s10461-024-04444-6)
Supplement: Supplementary file 1 — Supplementary Material 1 [file 10461_2024_4444_MOESM1_ESM.docx]

**Table S1**. Clinical and demographic information for visits in the first year after initiation of intervention. (For the three early sites in the Intervention Cohort, this is the 2016-2017 school year and for the three delayed sites in the Intervention Cohort, this is the 2017-2018 school year. For the Non-Intervention Cohort, this is taken to be the 2016-2017 school year.)

|  | **Intervention** | **Non- Intervention** |
| --- | --- | --- |
| n | 5883 | 3665 |
| Age (mean (SD)) | 15.87 (1.41) | 16.37 (1.53) |
| Gender (Male) (%) | 2430 (41.3) | 1510 (41.2) |
| Ethnicity (%) |  |  |
| Non-Span/Hisp/Lat | 1863 (31.7) | 1074 (29.3) |
| Span/Hisp/Lat | 2950 (50.1) | 1600 (43.7) |
| Unknown | 1070 (18.2) | 991 (27.0) |
| Race (%) |  |  |
| White | 198 ( 3.4) | 144 ( 3.9) |
| Black/Afr. Am. | 1512 (25.7) | 989 (27.0) |
| Other | 3410 (58.0) | 1813 (49.5) |
| Unknown | 763 (13.0) | 719 (19.6) |
| Sexually Active Ever (%) |  |  |
| No | 2745 (46.7) | 1398 (38.1) |
| Yes | 2492 (42.4) | 1860 (50.8) |
| NA | 646 (11.0) | 407 (11.1) |

**Table S2**. Clinical and demographic information for visits in the second year after initiation of intervention. (For the three early sites in the Intervention Cohort, this is the 2017-2018 school year and for the three delayed sites in the Intervention Cohort, this is the 2018-2019 school year. For the Non-Intervention Cohort, this is taken to be the 2017-2018 school year.)

|  | **Intervention** | **Non- Intervention** |
| --- | --- | --- |
| n | 5585 | 3705 |
| Age (mean (SD)) | 15.92 (1.39) | 16.31 (1.57) |
| Gender (Male) (%) | 2201 (39.4) | 1512 (40.8) |
| Ethnicity (%) |  |  |
| Non-Span/Hisp/Lat | 1635 (29.3) | 1003 (27.1) |
| Span/Hisp/Lat | 2809 (50.3) | 1543 (41.6) |
| Unknown | 1141 (20.4) | 1159 (31.3) |
| Race (%) |  |  |
| White | 167 ( 3.0) | 133 ( 3.6) |
| Black/Afr. Am. | 1368 (24.5) | 922 (24.9) |
| Other | 3098 (55.5) | 1729 (46.7) |
| Unknown | 952 (17.0) | 921 (24.9) |
| Sexually Active Ever (%) |  |  |
| No | 2270 (40.6) | 1298 (35.0) |
| Yes | 2463 (44.1) | 1816 (49.0) |
| NA | 852 (15.3) | 591 (16.0) |

**Table S3**. Clinical and demographic information for visits in the third year after initiation of intervention. (For the three early sites in the Intervention Cohort, this is the 2018-2019 school year and for the three delayed sites in the Intervention Cohort, this is the 2019-2020 school year. For the Non-Intervention Cohort, this is taken to be the 2018-2019 school year.)

|  | **Intervention** | **Non- Intervention** |
| --- | --- | --- |
| n | 5158 | 3949 |
| Age (mean (SD)) | 15.87 (1.39) | 16.22 (1.54) |
| Gender (Male) (%) | 2043 (39.6) | 1677 (42.5) |
| Ethnicity (%) |  |  |
| Non-Span/Hisp/Lat | 1436 (27.8) | 1147 (29.0) |
| Span/Hisp/Lat | 2587 (50.2) | 1729 (43.8) |
| Unknown | 1135 (22.0) | 1073 (27.2) |
| Race (%) |  |  |
| White | 148 ( 2.9) | 126 ( 3.2) |
| Black/Afr. Am. | 1199 (23.2) | 981 (24.8) |
| Other | 2833 (54.9) | 1830 (46.3) |
| Unknown | 978 (19.0) | 1012 (25.6) |
| Sexually Active Ever (%) |  |  |
| No | 2043 (39.6) | 1355 (34.3) |
| Yes | 2216 (43.0) | 1781 (45.1) |
| NA | 899 (17.4) | 813 (20.6) |

**Table S4**. Clinical and demographic information for visits in the fourth year after initiation of intervention. (For the three early sites in the Intervention Cohort, this is the 2019-2020 school year; note that the three delayed sites in the Intervention Cohort only received 3 years of intervention so, they are not included here. For the Non- Intervention Cohort, this is taken to be the 2019-2020 school year.)

|  | **Intervention** | **Non- Intervention** |
| --- | --- | --- |
| n | 2207 | 3124 |
| Age (mean (SD)) | 15.93 (1.38) | 16.19 (1.57) |
| Gender (Male) (%) | 876 (39.7) | 1249 (40.0) |
| Ethnicity (%) |  |  |
| Non-Span/Hisp/Lat | 580 (26.3) | 879 (28.1) |
| Span/Hisp/Lat | 900 (40.8) | 1402 (44.9) |
| Unknown | 727 (32.9) | 843 (27.0) |
| Race (%) |  |  |
| White | 56 ( 2.5) | 93 ( 3.0) |
| Black/Afr. Am. | 485 (22.0) | 751 (24.0) |
| Other | 993 (45.0) | 1419 (45.4) |
| Unknown | 673 (30.5) | 861 (27.6) |
| Sexually Active Ever (%) |  |  |
| No | 593 (26.9) | 999 (32.0) |
| Yes | 919 (41.6) | 1458 (46.7) |
| NA | 695 (31.5) | 667 (21.4) |
